# Job Satisfaction and Burnout among Intensive Care Unit Nurses and Physicians

**DOI:** 10.1155/2013/786176

**Published:** 2013-11-05

**Authors:** Hilde Myhren, Øivind Ekeberg, Olav Stokland

**Affiliations:** ^1^Intensive Care Unit, Oslo University Hospital, Ullevål, Oslo, Norway; ^2^Department of Acute Medicine, Oslo University Hospital, Ullevål, Oslo, Norway; ^3^Department of Behavioural Sciences in Medicine, Faculty of Medicine, University of Oslo, Oslo, Norway

## Abstract

*Introduction*. Nurses and physicians working in the intensive care unit (ICU) may be exposed to considerable job stress. The study aim was to assess the level of and the relationship between (1) job satisfaction, (2) job stress, and (3) burnout symptoms. *Methods*. A cross-sectional study was performed at ICUs at Oslo University Hospital. 145 of 196 (74%) staff members (16 physicians and 129 nurses) answered the questionnaire. The following tools were used: job satisfaction scale (scores 10–70), modified Cooper's job stress questionnaire (scores 1–5), and Maslach burnout inventory (scores 1–5); high score in the dimension emotional exhaustion (EE) indicates burnout. Personality was measured with the basic character inventory. Dimensions were neuroticism (vulnerability), extroversion (intensity), and control/compulsiveness with the range 0–9. *Results*. Mean job satisfaction among nurses was 43.9 (42.4–45.4) versus 51.1 (45.3–56.9) among physicians, *P* < 0.05. The mean burnout value (EE) was 2.3 (95% CI 2.2–2.4), and mean job stress was 2.6 (2.5–2.7), not significantly different between nurses and physicians. Females scored higher than males on vulnerability, 3.3 (2.9–3.7) versus 2.0 (1.1–2.9) (*P* < 0.05), and experienced staff were less vulnerable, 2.7 (2.2–3.2), than inexperienced staff, 3.6 (3.0–4.2) (*P* < 0.05). Burnout (EE) correlated with job satisfaction (*r* = −0.4, *P* < 0.001), job stress (*r* = 0.6, *P* < 0.001), and vulnerability (*r* = 0.3, *P* = 0.003). *Conclusions*. The nurses were significantly less satisfied with their jobs compared to the physicians. Burnout mean scores are relatively low, but high burnout scores are correlated with vulnerable personality, low job satisfaction, and high degree of job stress.

## 1. Introduction

Nurses and physicians working in the intensive care unit (ICU) may be exposed to considerable work related stress [1, 2], distress symptoms among staff have been described as being frequent [3, 4], and previous studies have identified high levels of burnout symptoms among ICU staff [5–10]. Burnout is a psychological concept often used as a response to long-term emotional and interpersonal stressors, usually in the work context. Burnout may be a result of too much work and too little recovery. The clinical impact of burnout may be decreased well-being (insomnia, irritability, eating problems, and depressive problems) and increased sick leave among staff [7]. A high degree of emotional exhaustion among nurses has been shown to predict lower self-rated performances and higher intention to quit work [11, 12].

Characteristics of both the organisation (decision makers, authority) and the work (workload, social support, and autonomy) may be associated with job satisfaction and psychological reactions like burnout in staff [13–15]. Previous ICU studies have identified several factors associated with the development of burnout: personal characteristics, working conditions, particularly in the form of long-term overload, ICU, and conflicts, little support and no feeling of useful work [5, 7, 9, 10, 16]. Conflicts were found to be frequent and associated with increased job strain [10, 17, 18]. Personality has also shown to influence problem-solving coping strategies and perceived job stress [19], and neuroticism was found to be a predictor of burnout, correlating with emotional exhaustion [20]. Physicians with extravert personality reported more personal accomplishment and were more satisfied [20]. It is therefore suggested that differences in approach to work and perceived job stress reflect stable personality differences among physicians themselves. However, knowledge about the relationship between personality and burnout among ICU staff is poor and has not previously been studied in Norwegian ICU nurses and physicians.

### 1.1. Aims of the Study

1. To assess job satisfaction, job stress, and the level of burnout among ICU nurses and physicians.
2. To assess if there is an association between burnout symptoms and
  - job satisfaction.
  - job stress.
  - personality traits.
  - professions, gender, experience, and department.

## 2. Methods

A cross-sectional study was performed at three ICUs at Oslo University Hospital, Ullevål. A six-bed medical ICU, an 11-bed general ICU, and a coronary unit with three beds for mechanically ventilated coronary patients participated in this study. All units have specially trained intensive care nurses with a nurse-bed ratio about 1 : 1. These units were organized in different departments with their own management. Nurses and physicians, permanently employed in the ICU, were invited to fill in a questionnaire. Staff on long-term sick leave (*n* = 6) or not at work due to maternity leave (*n* = 15) were not eligible to participate and were excluded. The questionnaire was delivered in the mailbox to eligible staff. One reminder was put in their mailbox, and they were reminded about the study several times by the study investigators during daily work. They answered the questionnaire anonymously and returned their reply in a box at the staff's resting room.

### 2.1. The Questionnaire

The questionnaire included measures of staff characteristics: department, gender, profession, and number of years working with ICU patients. In addition, four instruments were used to measure job satisfaction, job stress, burnout, and personality trait, respectively, Table 1.

1. The job satisfaction scale (JSS) consists of ten questions on various aspects of working conditions, working hours, responsibility, variation, collaborations, and salary [21]. All items are scored on a scale from 1 (extremely dissatisfied) to 7 (extremely satisfied) and combined in a sum score with range from 10 (low satisfaction) to 70 (high satisfaction). The questionnaire has been translated and used previously in Norway and found valid [22].
2. A modified version of the Cooper's job stress questionnaire (CJSQ) with 16 questions was used [23]. A similar version has been validated [24] and used previously in Norwegian studies [25]. Responses are given on a 5-point scale; higher score reflects high degree of stress (1 = no stress, 5 = very much stress). All item scores are combined in a sum score and then divided on the total number of items to provide an average score with range 1–5.
3. The Maslach burnout inventory (MBI) [28] is the most used instrument to measure symptoms of burnout. It has been validated for a variety of occupations. The translated Norwegian version of this questionnaire consists of 25 items. It is composed of three dimensions: emotional exhaustion (EE), 8 items, depersonalisation (DP), 4 items, and personal accomplishment (PE), 7 items. Six questions are filler items and are not used in the sum score. As in previous studies of Norwegian physicians [24, 26], we used a five-point Likert scale (1 = does not fit, 5 = fits very well) as the original frequency scale has been criticised for having categories that are not mutually exclusive [24]. Items in each dimension are combined in a sum score and then divided on the number of items in that dimension, range 1–5. Higher scores on EE and DP and lower scores on PE indicate burnout.
4. To measure personality, the basic character inventory (BCI) was used. This instrument is composed of three dimensions: neuroticism (vulnerability), extroversion (intensity) and control/compulsiveness [27]. The vulnerability dimension closely resembles neuroticism and includes questions about sensitivity to other people's opinions and criticism. The intensity dimension is a measure of extraversion/introversion, and the control dimension describes the degree of compulsiveness. Each dimension is based on nine questions with a dichotomous response, giving a range from 0 (low) to 9 (high).

### 2.2. Statistics

Statistical analyses were performed with the SPSS for Windows Version 15.0. Continuous variables are presented with mean score with 95% confidence intervals (CI). The significance level was set at *P* < 0.05. Independent sample *t*-test was used when comparing two groups on normally distributed variables. Correlations between pairs of continuous variables were calculated using Pearson's correlation coefficients. To identify variables independently associated with burnout, linear regression analyses were performed. Variables that were associated with burnout in the bivariate analyses (*P* < 0.5) were included in a multivariate regression model. All independent variables included in the multivariate regression analyses correlated below 0.7. We further compared scores between experienced and inexperienced medical staff divided on the median of number of years working in an ICU (median = 7 years). Staff who were experienced (*n* = 78) were defined as having worked seven or more years in an ICU, and inexperienced staff had worked less than seven years.

### 2.3. Ethics

The regional ethics committee approved the study.

## 3. Results

A total of 196 nurses and physicians were invited to participate. Among these, 145 (74%) answered the questionnaire, 129 were nurses, and 16 were physicians (Table 2). The response rates of physicians and nurses were not significantly different, 80% versus 73%. Most participants were females (84%). The mean number of years working in the ICUs was 8.8 (95% CI: 7.8–9.7). The number of staff responders from the 11-bed general ICU was 76 (52%), from the six-bed medical ICU was 39 (27%), and from the three-bed coronary care unit was 30 (21%).

### 3.1. Differences between Professions: Nurses and Physicians

Table 2 shows staff characteristics, job satisfaction, job stress, burnout, and personality trait scores for all participants and divided into nurses and physicians. There were significantly more females among nurses compared to physicians, 87% versus 25%, *P* < 0.001. No differences between the professions were seen in regard to experience, job stress, or burnout scores. Physicians scored significantly higher on job satisfaction compared to the nurses, 51.1 versus 43.9, *P* < 0.05, and the nurses scored significantly higher on the personality trait control compared to the physicians, 3.9 versus 2.9, *P* < 0.05. 

### 3.2. Gender Difference, Differences due to Experience

No differences between genders or due to experience were seen with regard to job satisfaction, job stress, or burnout scores (Table 2). Only the personality trait neuroticism (vulnerability) was significantly different between genders as females scored higher compared to males, 3.3 (95% CI 2.9–3.7) versus 2.0 (95% CI 1.1–2.9), *P* < 0.05. Nurses and physicians who were experienced, defined as having worked seven or more years in an ICU, scored significantly lower on the personality trait neuroticism (vulnerability) compared to inexperienced staff, 2.7 (95% CI 2.2–3.2) versus 3.6 (95% CI 3.0–4.2), *P* < 0.05 (not shown in table).

### 3.3. Differences between Departments

Finally, we compared scores between the different departments. No differences were seen between the departments in regard to burnout score, job stress, or experience among the staff. However, staff in the six-bed medical ICU scored significantly higher on job satisfaction, 49.5 (95% CI 47.1–51.9), compared to the general ICU, 42.7 (95% CI 40.7–44.7), and the coronary ICU, 43.5 (95% CI 40.3–46.6), *P* < 0.01. Some differences in patient characteristics between the three departments were seen: more patients were male in the cardiac ICU (84% versus 61% in the general ICU and 58% in the medical ICU, *P* < 0.05). The general ICU and cardiac ICU had significantly more patients on mechanical ventilation (95% and 100% versus 62% in the medical ICU, *P* < 0.05), and the general ICU had longer duration of mechanical ventilation (14 days versus seven days in the medical ICU and eight days in the cardiac ICU, *P* < 0.05). Trauma and patients with head injury were only treated in the general ICU. However, no significant differences in patient age, length of ICU stay,) simplified acute physiology score (SAPS), and nine equivalents in measuring nurses manpower use score (NEMS) were seen.

### 3.4. Associations between Burnout and Job Satisfaction, Job Stress and Personality

Staff with high degree of job satisfaction and staff with low degree of job stress scored significantly lower on the burnout dimensions depersonalisation (DP) and emotional exhaustion (EE) as shown in Table 3. The personality trait neuroticism (vulnerability) was significantly associated with the two burnout dimensions EE and DP, and the personality trait control was associated with DP. No associations were seen with the dimension personal accomplishment. Analyses between burnout and staff characteristics, profession, gender, experience, and department, showed no significant correlations (not shown in the table).

### 3.5. Predictors of Burnout

To explore factors associated with the burnout dimension emotional exhaustion (EE), several variables were analyzed: profession, gender, experience, department, personality, job satisfaction and job stress (Table 4). In the bivariate analyses, the personality dimension neuroticism (vulnerability), job satisfaction, and job stress were significantly associated with burnout (EE). In the multivariate regression analyses, job satisfaction and job stress were found to be independent predictors of the burnout dimension EE in this model. Explained variance in the model was *r* ^2^ = 0.39. Multivariate analyses with burnout dimension depersonalisation as the dependent variable were performed, and job satisfaction (beta –0.02, *P* = 0.002), job stress (beta 0.20, *P* = 0.008), and personality dimension control (beta 0.05, *P* = 0.016) were found to be predictors. No variables were significantly associated with burnout dimension personal accomplishment. 

## 4. Discussion

The ICU is a highly stressful environment, not only for patients and relatives but also the medical staff. Difficult decisions about end-of-life care are made, and burnout is found to be frequent among ICU staff [4, 6, 7, 10]. Increased knowledge about job satisfaction and burnout is important because this may affect quality of patient care, poor communication with relatives, and high staff turnover rates [18]. In this cross-sectional study, burnout, job satisfaction, and perceived job stress in Norwegian ICU nurses and physicians were explored. 

Even though people who experience all three dimensions of burnout have the greatest degree of burnout, emotional exhaustion has been used as the main hallmark [28]. Single item measures of emotional exhaustion and depersonalisation were also found to be useful for assessing burnout in medical professionals [29]. Comparison with burnout scores in other ICU studies is difficult because different versions of the MBI were used [6, 7, 9]. To compare our results with those of Guntupalli et al., a study of burnout including the internal medicine intensivists (physicians) was performed we multiplied the mean score with the number of items in each dimension. The study of Guntupalli et al. was performed at medical ICUs at large hospitals, and nearly 90% of the respondents were males. The mean score on emotional exhaustion in our study was 18.4 versus 22.2 in Guntupalli et al.'s study, whereas depersonalisation was 6.4 in our study versus 7.1 in their study and personal accomplishment 24.5 in our study versus 30.9 in their study. It therefore seems likely that our ICU staff, nurses, and physicians suffer less from burnout symptoms compared to the study of Guntupalli et al. There may be several explanations for the lower level of burnout in our study compared to Guntupalli et al. The participants in their study were all physicians and more often male. The participants did also work halftime outside the ICU, and only internal medical physicians were included. In addition, the study was presented 17 years ago, with a poor social support system.

In contrast to previous studies that found more burnout in females [8] and more burnout in physicians [6, 7, 10], we found no gender or profession differences. Another study found less burnout symptoms in females [14]. However, this study is the first to show that ICU physicians showed significantly higher degree of job satisfaction compared to ICU nurses. In comparison with previous Norwegian studies, the level of job satisfaction among ICU physicians is similar to satisfaction in Norwegian general practitioner [22, 30] and primary care team members [31]. We have reason to believe that the low satisfaction among ICU nurses may affect their performance and patient care. Several factors like autonomy and workload may be associated with job satisfaction. Physicians may have more autonomy and have a greater impact on patient related decisions than nurses in general. Working hours, number of nightshifts, nurse-to-bed ratio, organization, and relation to the unit leader may be different. However, this study is unable to explain the differences in job satisfaction found among nurses and physicians. The job satisfaction between the three units was also significantly different. The units differ in patient diagnoses, frequency and duration of mechanical ventilation, management, and location. The workload and nightshifts may also be different but was not registered for this study.

Even though this study found that low job satisfaction and high job stress were predictors of burnout (EE), causality is difficult to prove, particularly in a cross-sectional study. However, it is likely that burnout has multiple causes and job satisfaction, job stress, and vulnerable personality may be important factors. 

### 4.1. Limitations

 The Maslach burnout inventory is the most studied instrument to measure symptoms of burnout and can be used as an indicator of the syndrome. Even though it correlates highly with other measures of burnout, the correlation with a clinical evaluation of burnout may be low. Structured clinical interviews should be performed in addition to the MBI, and further investigation of personality is needed. Using the Norwegian version made comparisons with international studies difficult. More experienced staff were less vulnerable in the present study; however, this may partly be biased by selection if vulnerable staff drop out of ICU work. Staff on long-term sick leave (*n* = 6) may have suffered from burnout and biased the study. The cross-sectional design of this study makes conclusion in regard to causality difficult. With the limited number of physicians and males, significant differences between professions or genders might have been missed. The single-center design of the present study could be seen as a limitation and may suggest the need of multicenter studies as most ICUs have limited numbers of physicians and males [14]. 

## 5. Conclusion

The nurses were significantly less satisfied with their job compared to the physicians. Burnout mean scores were relatively low, but higher burnout scores were correlated to the personality trait vulnerability, low job satisfaction, and high degree of job stress. The job satisfaction between the three ICU units was significantly different. The reason for this is unknown but calls for studies that investigate differences in both patients' diagnoses and ICU management in relation to burnout among ICU staff.

## Figures and Tables

**Table 1:** Description of the instruments.

Dimension	Range	Range of response alternatives	Items (*n*)	Cronbach's alpha
Burnout (MBI)^1^				
Emotional exhaustion	1–5	Does not fit–fits very well	8	0.70
Depersonalisation	1–5	Does not fit–fits very well	4	0.70
Personal accomplishment	1–5	Does not fit–fits very well	7	0.42
Job satisfaction	10–70	Extremely dissatisfied-extremely satisfied	10	0.85
Cooper's job stress questionnaire	1–5	No stress–very much stress	16	0.85
Personality (BCI)^2^				
Neuroticism (vulnerability)	0–9	Does not fit–fits	9	0.71
Extroversion (intensity)	0–9	Does not fit–fits	9	0.76
Control	0–9	Does not fit–fits	9	0.54

^1^MBI: Maslach burnout inventory, ^2^BCI: basic character inventory.

**Table 2:** Staff characteristics. Differences between professions and gender.

	All	Nurses	Physicians		Male	Female	
	*N* = 145	*N* = 129	*N* = 16		*N* = 23	*N* = 122	
Females	84%	87%	25%	**∗∗**			
Mean years in ICU	8.8 (7.8–9.7)	8.8 (7.8–9.8)	7.6 (4.3–10.9)	ns	7.5 (5.4–9.5)	8.9 (7.8–10.0)	ns
Burnout (MBI)^1^							
Emotional exhaustion	2.3 (2.2–2.4)	2.3 (2.2–2.4)	2.2 (2.0–2.4)	ns	2.2 (2.0–2.4)	2.3 (2.2–2.4)	ns
Depersonalisation	1.6 (1.5–1.7)	1.6 (1.5–1.7)	1.4 (1.2–1.6)	ns	1.6 (1.4–1.8)	1.6 (1.5–1.7)	ns
Personal accomplishment	3.5 (3.4–3.5)	3.5 (3.4–3.6)	3.4 (3.2–3.6)	ns	3.4 (3.3–3.5)	3.5 (3.4–3.6)	ns
Job satisfaction	44.7 (43.3–46.1)	43.9 (42.4–45.4)	51.1 (45.3–56.9)	**∗**	44.6 (40.3–48.9)	44.3 (42.7–45.9)	ns
Job stress	2.6 (2.5–2.7)	2.6 (2.5–2.7)	2.7 (2.3–3.1)	ns	2.5 (2.2–2.7)	2.6 (2.5–2.7)	ns
Personality (BCI)^2^							
Neuroticism	3.1 (2.7–3.4)	3.1 (2.7–3.5)	2.8 (1.8–3.8)	ns	2.0 (1.1–2.9)	3.3 (2.9–3.7)	**∗**
Extroversion	5.7 (5.3–6.2)	5.7 (5.2–6.1)	6.1 (4.7–7.4)	ns	5.7 (4.6–6.8)	5.7 (5.2–6.2)	ns
Control	3.8 (3.5–4.1)	3.9 (3.6–4.2)	2.9 (1.7–4.1)	**∗**	4.2 (3.2–5.2)	3.8 (3.5–4.2)	ns

Mean values with 95% confidence intervals.

^1^MBI: Maslach burnout inventory, ^2^BCI: basic character inventory.

**P < 0.001, *P < 0.05.

**Table 3:** Associations between burnout and job satisfaction, job stress, and personality.

	Burnout (MBI)^1^		
	Emotional exhaustion	depersonalisation	Personal accomplishment
Job satisfaction			
Pearson's corr.	**−0.414**	**−0.313**	0.122
Sig. (2-tailed)	**<0.001**	**<0.001**	0.145
Cooper's job stress quest.			
Pearson's corr.	**0.586**	**0.293**	0.105
Sig. (2-tailed)	**<0.001**	**<0.001**	0.208
Personality (BCI)^2^			

Neuroticism			
Pearson's corr.	**0.274**	**0.231**	0.009
Sig. (2-tailed)	**0.001**	**0.005**	0.919
Extroversion			
Pearson's corr.	−0.070	−0.099	0.077
Sig. (2-tailed)	0.405	0.241	0.362
Control			
Pearson's corr.	0.136	**0.179**	−0.020
Sig. (2-tailed)	0.107	**0.033**	0.815

^1^MBI: Maslach burnout inventory, ^2^BCI: basic character inventory.

Bold data refer to significant values in the analyses (*P* < 0.05).

**Table 4:** Predictors of the burnout dimension emotional exhaustion.

	Bivariate			Multivariate		
	Beta value	CI	*P* value	Beta value	CI	*P* value
Profession^1^	−0.08	−0.33–0.18	0.560			

Gender^2^	0.13	−0.09–0.35	0.259			
Experience^3^	−0.00	−0.02–0.01	0.757			
Department^4^						
Medical	−0.22	−0.40 to −0.03	0.021			
Cardiac	−0.07	−0.27 to −0.13	0.479			
Job satisfaction	**−0.02**	**−0.03 to −0.01**	**<0.001**	**−0.01**	**−0.02 to −0.01**	**<0.001**
Job stress	**0.50**	**0.39**–**0.62**	**<0.001**	**0.43**	**0.32**–**0.55**	**<0.001**
Personality (BCI)^5^						
Neuroticism	**0.06**	**0.03–0.10**	**0.001**			
Extroversion	−0.01	−0.05–0.02	0.405			
Control	−0.03	−0.01–0.07	0.107			

Bivariate and multivariate linear regression analyses of variables associated with the burnout dimension emotional exhaustion.^ 1^Profession: 1: nurse, 2: physician, ^2^Gender: 1: male, 2: female, ^3^Experience: 1: inexperienced, 2: experienced, ^4^Department: 1: general ICU, 2: medical ICU, and 3: cardiac ICU, and ^5^BCI: basic character inventory.

Bold data refer to significant values in the analyses (*P* < 0.05).
